# Potential Regulatory Role of miR-21 on Alzheimer’s Disease by Targeting GSK-3β Signaling

**DOI:** 10.31661/gmj.v12i.3027

**Published:** 2023-05-13

**Authors:** Haojun Ding

**Affiliations:** ^1^ Henan Vocational College of Industry and Trade, Zhengzhou 450000, China

**Keywords:** Alzheimer’s Disease, miR-21, PI3K/AKT/GSK-3β, Apoptosis

## Abstract

Background: Alzheimer’s disease (AD) is the most important neurogenerative disorder with progressive dementia as its main clinical manifestation. The microRNAs (miRNAs) are identified as crucial modulators in AD progression. Nevertheless, the biological potential of miR-21 in AD is obscure. Hence, this study aimed to evaluate the possible role of miR-21 in the pathogenesis of AD via phosphatidylinositol-4,5-bisphosphate 3-kinase (PI3K)/protein kinase B (AKT)/glycogen synthase kinase-3beta (GSK-3β) signaling.Materials and Methods: The miR-21 expression in the brain tissues of patients with AD, as well as normal brain tissues and Aβ1-42-stimulated SH-SY5Y cell line (AD model) was examined by in situ hybridization and quantitative real-time polymerase chain reaction. Also, the apoptosis-linked protein levels as well as programmed cell death 4 (PDCD4) were detected by western blot. Results: Our findings revealed that miR-21 was low expressed in the brain tissues of patients with AD and AD model (P0.01). Also, the miR-21 overexpression could inhibit apoptosis of the AD model (P0.01). Indeed, the miR-21 negatively regulated PDCD4 expression, which led to activated PI3K/AKT/GSK-3β signaling.Conclusion: Our study demonstrated that miR-21 cloud inhibits cell apoptosis in AD through the activation of PI3K/AKT/GSK-3β signaling pathway using inhibition of PDCD4 expression.

## Introduction

As the most frequent type of neurodegenerative disorder, Alzheimer’s disease (AD) is mainly manifested by impairment in cognition, learning disabilities, decreased ability to independent living, and other symptoms that seriously affect social and occupational life [1].

Genetic and environmental factors influence the initiation and development of AD; age and an unhealthy lifestyle could also increase the risk of disease [2].

Its pathogenesis consists of neuronal dysplasia, altered levels of angiogenic biomarkers, and the production of inflammatory mediators [3][4].

MicroRNAs (miRNAs) refer to evolutionarily conserved short noncoding transcripts, which induce degradation and translational suppression of target miRNAs via combining with their 3’ untranslated region (3’UTR) [5].

Previous evidence reveals that miRNA dysregulation is correlated with AD progression [6], e.g., elevated miR-34c affects synaptic deficits via the reactive oxygen species (ROS)-JNK-p53 pathway [7].

Also, the miR-126a-3p could affect hippocampal memory via AD-related proteins [8]. Evidence showed that miR-21, a key member of miRNAs, is involved in the progression of various diseases, such as lung cancer [9], ischemic neuronal injury [10], and cardiorenal syndrome [11].

Moreover, miR-21 accelerates nerve growth factor signal transduction and modulates neuronal degeneration in the PC12 cell line [12].

The phosphatidylinositol-4,5-bisphosphate 3-kinase (PI3K)/protein kinase B (AKT) signaling pathway has a crucial impact on neuroinflammation [13] and belongs to a heteromeric protein containing a p110 subunit together with a p85 subunit [14]. PI3K p110 subunits shift PIP2 into PIP3, which in reverse stimulates AKT [15]. Glycogen synthase kinase-3β (GSK-3β) belongs to a downstream kinase of the PI3K/AKT signaling and is also a constitutively active protein kinase along with various biological potentials containing neuroinflammation as well as accumulation of neurofibrillary tangles [16]. Upon activation, GSK-3β facilitates microglial migration as well as inflammatory activation by increasing the production of pro-inflammatory cytokines [17].

As reported previously, GSK-3β is also involved in cell apoptosis, cell differentiation, embryonic development, neurogenesis, neuronal polarization, and axon dendrite morphological regulation [18]. Evidence suggests that GSK-3β is drawn in modulating mental diseases and neurodegenerative disorders and is also a key element in various signal cascade reactions [19].

In patients with AD, the activity of GSK-3β increases, resulting in abnormal hyperphosphorylation of TAU protein [20].

Also, the activation of GSK-3β could inhibit the secretory cleavage of amyloid precursor protein and increase the production of a β42 [21].

Hyperphosphorylated TAU and oligomer β42 are the main causes of neuronal dysfunction and cognitive impairment in the early stage of AD [22]; however, the interaction mechanism in the pathogenesis is obscure.

Hence, the present study aimed to investigate the effect of miR-21 on the apoptosis of AD via targeting the PI3K/AKT/GSK-3β signaling pathway.

## Materials and Methods

**Table:** Table1. Sequences of Investigated Genes Primers for Gene Expression Detection

**Genes**	**Primer sequence**
**miR-21**	F: 5’-GGGGTAGCTTATCAGACTG-3’
R: 5’-TGGAGTCGGCAATTGCACTG-3’	
**GSK-3β**	F: 5’-GGGATGGGCACTGAAATA-3’
R: 5’-CATTTCGGCAGACAATACAA-3’	
**U6**	F: 5’-GGATCAATACAGAGCAGATAAGC-3’
R: 5’-CTTTCTGAATTTGCGTGCC-3’	
**GAPDH**	F: 5’-CCCACTCCTCCACCTTTGAC-3’
R: 5’-CATACCAGGAAATGAGCTTGACAA-3’	

**GSK-3β:** Glycogen synthase kinase-3beta,**GAPDH:**Glyceraldehyde-3-phosphate dehydrogenase

1. Samples and Cell Culture

Totally 20 brain tissue samples of patients with AD and ten normal brain tissue samples were obtained. Also, SH-SY5Y cells were provided by American Type Culture Collection (ATCC, USA) and incubated in Dulbecco’s modified Eagle medium (Gibco, USA) containing 1% penicillin/streptomycin (Thermo Fisher, USA) together with 10% fetal bovine serum (Thermo Fisher, USA) at 37°C, with 5% CO2. To induce AD in vitro model, Aβ1-42 peptide (Sigma, USA) was dissolved at a concentration of 1 mM, followed by treatment into SH-SY5Y cells.

2. miR-21 Expression Assessment

2.1. In Situ Hybridization (ISH)

To evaluation of miR-21 expression in brain tissue samples, ISH was performed according to the kit protocol (Wuhan, China). Briefly, the tissue sections were pre-hybridized for 2 hours at 37 °C, then hybridized with DIG-labelled miR-21 probes overnight. Afterwards, the sections were stained with diaminobenzidine and hematoxylin (Beyotime, China). Then, all the sections were examined using the Olympus fluorescence microscope (Olympus America, Melville, NY, USA).

2.2. Cell Transfection

The miRNA mimics and anti-miRNA of miR-21 were obtained from Beijing Taitianhe Biotechnology Co., Ltd. (Beijing, China). SH-SY5Y cells were placed in 6-well plates at 60% confluence and incubated for 24 hours. Then, miRNA simulation and anti-miRNA transfection were performed with Lipofectamine 2000 transfection reagent (Millipore, USA).

2.3. RNA Isolation and Quantitative Real-Time Polymerase Chain Reaction (q-RT-PCR)

Trizol reagent (Beyotime, Shanghai, China) was adopted for total RNA isolation. The TaqMan™ Advanced miRNA cDNA Synthesis Kit (Thermo Fisher, USA) and First-Strand qRT-PCR kit (Invitrogen, USA) were applied for cDNA synthesis. PCR was performed by SYBR Green PCR Master Mix (Thermo Fisher, USA).

The 2-∆∆CT method was implemented to evaluate the relative levels of genes. The U6 and GAPDH were used as the internal control. The primer sequences used in this study are presented in Table-1.

3. Western Blot Analysis

Cells were incubated with RIPA lysis buffer (Thermo Fisher, USA), treated with protease on ice for 15 minutes, and separated at 12000 rpm at 4°C for 15 minutes. The BCA method was applied to estimate the protein concentration of samples. Mix the sample with the loading buffer and boil for 10 minutes. The protein was loaded on 12% SDS-PAGE and then shifted into the PVDF membrane (Millipore, USA). After sealing with 5% BSA, the membrane was incubated at 4℃ overnight with a specific primary antibody. The primary antibodies, including Bcl-2 (1/1000, Abcam, UK), Bax (1/1000, Abcam, UK), programmed cell death 4 (PDCD-4) (1/1000, Abcam, UK), PI3K (1/2000, Abcam, UK), p-PI3K (1/1000, Abcam, UK), AKT (1/2000, Abcam, UK), p-AKT (1/1000, Abcam, UK), GSK-3β (1/2000, Abcam, UK), and GAPDH (1/1000, Abcam, UK) were used. After washing, the membrane was further cultured with horseradish peroxidase bound anti-rabbit/mouse IgG (1/5000, Abcam, UK) for 2 hours. After secondary antibody incubation, the membrane was rinsed, and an enhanced chemiluminescence substrate (Thermo Fisher, USA) was used for immunoreactive band visualization. The results were collected and quantified by NIH ImageJ software.

4. Ethical Considerations

This study was approved by the ethics committee of Henan Vocational College of Industry and Trade (approval code: ZZU(H)2021-0017). Also, the written informed consent was obtained from all patients.

5. Statistical Analysis

Data analysis was performed using GraphPad 8.0 software (GraphPad Software Inc., USA). Also, t-test and one-way ANOVA were applied. All experiments were conducted in triplicate, and all results were presented as the mean ± SD. A P<0.05 was considered as significant difference.

## Results

**Figure-1:**
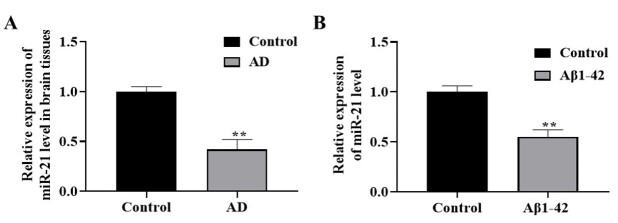
Figure 1. The expression level of miR-21. A: ISH analysis revealed low expression of miR-21 in the brain tissues of patients with AD. Also, qRT-PCR findings (B) indicated miR-21 expression in the AD model is lower than in the untreated (control) group. **P<0.01 vs. control

miR-21 Expression in AD Brain Tissue Samples and SH-SY5Y Cells

The miR-21 expression in the brain tissues of patients with AD was assessed by the ISH analysis. Relative to normal brain tissues, miR-21 was downregulated in the brain tissues of patients with AD (Figure-1A). Besides, qRT-PCR displayed that miR-21 expression significantly declined in Aβ1-42-stimulated SH-SY5Y cells (AD model) relative to the untreated group (P<0.01, Figure-1B).

miR-21 Inhibits Cell Apoptosis

**Figure-2:**
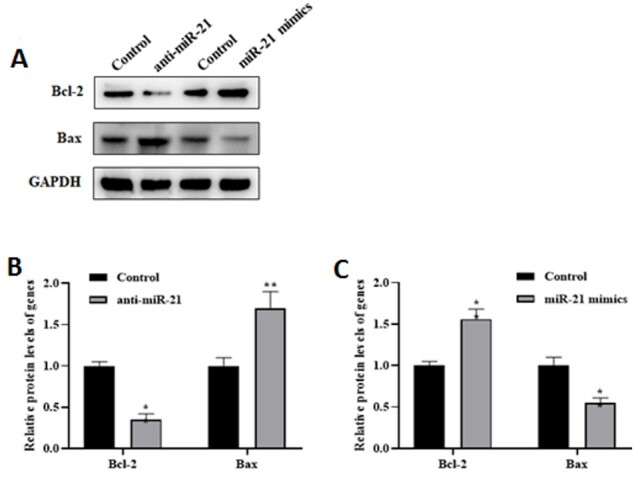
Figure 2. The miR-21 could inhibit the apoptosis of the AD model. Western blot analysis (A) showed the levels of Bcl-2 and Bax in AD model transfected with anti-miR-21 or miR-21 mimics. The result indicated that low expression (B) of miR-21 leads to down- and upregulated Bcl-2 and Bax protein levels, respectively. However, by increased the miR-21 level (C), the pro-apoptotic Bax protein level was reduced compared to the control level. The **P<0.01 vs. control

To evaluate the effect of miR-21 on apoptosis in the AD model, changes in apoptosis-associated proteins such as Bcl-2 and Bax were measured (Figure-2A). As shown in Figure-2B, miR-21 low expression resulted in the pro-apoptotic Bax protein level was upregulated, while the anti-apoptotic Bcl-2 protein level was decreased.

Inversely, after miR-21 overexpression, the Bax protein level was reduced, and the Bcl-2 protein level was elevated in the AD model compared to the control group (P<0.01, Figure-2C).

miR-21 Negatively Regulates PDCD4

**Figure-3:**
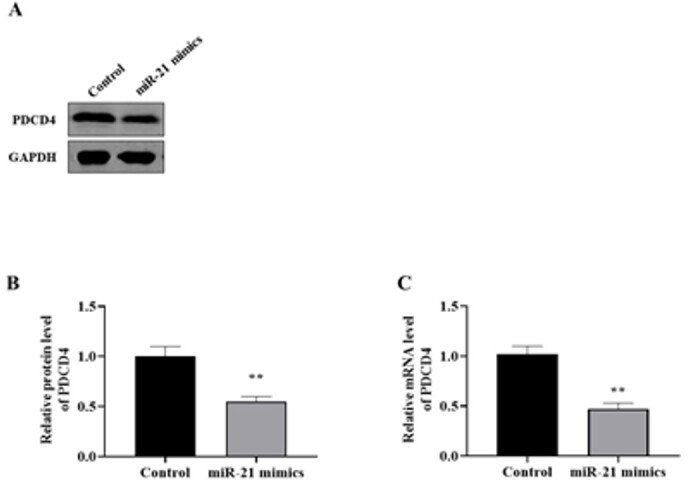
Figure 3. The miR-21 negatively regulates PDCD4. Western blot analysis (A and B) indicated PDCD4 protein levels decreased in AD model after miR-21 mimics transfection. Also, qRT-PCR (C) revealed PDCD4 mRNA levels after miR-21 mimics transfection significantly was reduced in AD model compared to the control group. **P<0.01 vs. control

Regarding western blot results, miR-21 overexpression could significantly downregulate PDCD4 protein levels (Figure-3A-B). In addition, qRT-PCR showed that PDCD4 mRNA expression was significantly inhibited by upregulating miR-21 (Figure-3C). Indeed, increased miR-21 levels could induce apoptosis in AD cells via the reduction of PDCD4 protein.

miR-21/PDCD4 Inhibits AD Cell Apoptosis Via PI3K/AKT/GSK3β Signaling Pathway

**Figure-4:**
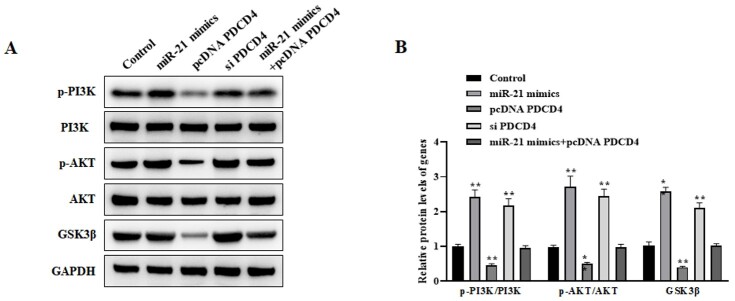
Figure 4. The miR-21/PDCD4 inhibits apoptosis via the PI3K/AKT/GSK3β signaling pathway. Western blot (A and B) assessed the levels of p-PI3K, p-AKT, and GSK3β in AD model transfected with miR-21 mimics, pcDNA PDCD4, si PDCD4, and miR-21 mimics+pcDNA PDCD4, respectively. **P<0.01 vs. control

Our findings indicated that PI3K/AKT/GSK3β signaling was activated by overexpressing miR-21 in AD model; however, PDCD4 overexpression showed the inhibitory effect on PI3K/AKT/GSK3β signaling. Also, miR-21 mimics and pcDNA PDCD4 co-transfection could reverse the activated PI3K/AKT/GSK-3β signaling induced by miR-21 mimics (Figure-4).

## Discussion

Dementia is the most common symptom of AD, which leads to disorientation and loss of memory and visual-spatial capacities in older adults [23][24]. However, neuropathology in the brains of AD patients occurred many years before these symptoms [25]. Hence, it is crucial to develop novel as well as effective biomarkers for AD diagnosis. Currently, miRNAs arise as novel therapeutic targets for various diseases, such as neuro-cognition disorders, cardiovascular diseases, and malignancies [26]. In this study, we evaluated the role of miR-21 and its relations with PDCD4 as well as the PI3K/AKT/GSK3β signaling pathway on regulating neuron apoptosis.

Previous reports revealed that miRNAs expressed in the brain tissue, and some are involved in neuron differentiation and memory formation [27].

Also, some miRNAs have been identified to downregulated in AD, such as miR-204-3p [8], miR-22-3p [29], and miR-124 [30]. Our research showed that miR-21 was also expressed in both the brain tissues of patients with AD and AD model, which suggested that miR-21 might play an inhibitory role in AD progression.

Some previous reports revealed that overexpressed miR-21 could inhibit cell apoptosis in various diseases [31][32]. Our study revealed that after miR-21 upregulation, the Bax protein level was decreased, while the Bcl-2 protein level was significantly elevated in the AD model, which indicates its anti-apoptotic properties. Similarly, Xu et al. demonstrated that the upregulation of miR-21 could also inhibit neuronal apoptosis [33].

PDCD4 is an important regulator of cell apoptosis [34]. Xiao et al. demonstrated that miR-21/PDCD4 axis has an anti-apoptotic role against cell death [35]. Besides, Cheng et al. reported that miR-21 protects cardiomyocytes against H2O2-induced cell apoptosis via targeting PDCD4 [36].

Our findings indicated that miR-21 could negatively regulate PDCD4 expression in AD model, which provides the inhibitory role of miR-21 on cell apoptosis via regulating PDCD4 expression.

The PI3K/AKT/GSK3β signaling pathway belongs to critical molecular signal transduction associated with various diseases by regulating biochemical and cytopathological processes [37].

Also, this pathway is important for neuronal survival with synaptic plasticity during neurodegenerative diseases, including AD [38].

In addition, GSK3-β is a crucial modulator in AD progression because dysregulated GSK3-β affects the main features of AD, containing tau phosphorylation, amyloid-β production, and neurogenesis along with synaptic function [39]. Of note, miR-21 is widely reported as an important factor in the progression of diseases via promoting the PI3K/AKT/GSK3β signaling [40][41].

Also, the current study demonstrated that miR-21 overexpression led to increased p-PI3K, p-AKT, and GSK3β protein levels, indicating that miR-21 overexpression promoted the PI3K/AKT/GSK3β signaling. Also, we showed that the activated PI3K/AKT/GSK3β signaling following miR-21 overexpression could reverse after co-transfection of pcDNA PDCD4, which proved that miR-21impact its role via inhibition of PDCD4 expression.

## Conclusion

Our study indicated that miR-21 was downregulated in AD, and via activating the PI3K/AKT/GSK3β signaling pathway could inhibit apoptosis in the AD model using inhibition of PDCD4 expression.

## Conflict of Interest

The authors declare no competing interests.
